# Advances in vulvovaginal candidiasis research: a comprehensive review from epidemiology, diagnosis, treatment to resistance mechanisms

**DOI:** 10.3389/fmicb.2026.1811011

**Published:** 2026-05-15

**Authors:** Weipei Zhu, Liangsheng Guo

**Affiliations:** Department of Obstetrics and Gynecology, The Second Affiliated Hospital of Soochow University, Suzhou, China

**Keywords:** biofilm, diagnosis, non-*albicans candida*, resistance mechanisms, treatment, vulvovaginal candidiasis

## Abstract

Vulvovaginal candidiasis (VVC), predominantly caused by *Candida albicans*, is one of the most common vaginal infections in women of reproductive age. Its clinical management has become increasingly complex due to the rising prevalence of non-*albicans Candida* infections, escalating azole resistance, and the challenge of biofilm formation. This review systematically summarizes recent advances in VVC research, with a focus on evolving epidemiology, innovations in diagnostics, current and emerging therapies, and an in-depth analysis of resistance mechanisms. Key molecular pathways underpinning antifungal resistance—including biofilm development, efflux pump overexpression, target-site gene mutations, and alterations in transcriptional regulators—are examined. The findings of this review support several actionable strategies for future practice. First, routine use of molecular diagnostics (PCR, MALDI-TOF MS) is essential for species identification and resistance detection, enabling a shift from empirical to precision-based therapy. Second, antifungal susceptibility testing should be interpreted with attention to vaginal pH conditions, and non-azole alternatives (boric acid, nystatin, ibrexafungerp, oteseconazole) should be prioritized when azole resistance is confirmed or suspected. Third, emerging approaches—including biofilm-disrupting agents, probiotic microbiome modulation, and nanotechnology-enhanced drug delivery—offer promising adjunctive and preventive strategies, particularly for recurrent VVC. By integrating these contemporary findings, this review provides a translational framework to optimize diagnosis, guide therapeutic decision-making, and inform future research priorities in VVC management. Notably, several novel agents—including ibrexafungerp and oteseconazole—have already received FDA approval and are entering clinical practice, with multiple ongoing trials evaluating boric acid, probiotics, and novel oral antifungals, underscoring the accelerating translation of mechanism-informed therapies into patient care.

## 1 Introduction

Vulvovaginal candidiasis (VVC) is a prevalent fungal infection, affecting a substantial majority of women globally during their reproductive years. Epidemiological studies suggest that 70–75% of women experience at least one episode of VVC in their lifetime, with 5–9% suffering from recurrent infections (RVVC), defined as three or more symptomatic episodes within a 12-month period (Foxman et al., 2000; Sobel, 2007). While *Candida albicans* remains the primary causative agent, a significant epidemiological shift has been observed: non-*albicans Candida* (NAC) species—notably *Nakaseomyces glabratus (formerly Candida glabrata)*, *Pichia kudriavzevii* (formerly *C. krusei*), *C. tropicalis*, and *C. parapsilosis*—are increasingly prevalent. This trend poses a mounting clinical challenge, as many NAC species exhibit inherent or acquired resistance to first-line antifungal therapies (Parazzini et al., 2000; Richter et al., 2005; Kennedy and Sobel, 2010; Makanjuola et al., 2018). However, directly comparing prevalence estimates across studies remains challenging due to significant methodological heterogeneity. For instance, studies relying solely on culture-based methods may underestimate true prevalence compared to those employing molecular techniques like PCR, which detect lower fungal burdens. Furthermore, estimates vary substantially depending on whether the study population comprises symptomatic women attending clinics (where prevalence is higher) or asymptomatic women in community settings (where colonization is often reported). These methodological distinctions are critical when interpreting regional differences, such as the higher NAC prevalence reported in studies from Africa and the Middle East compared to Europe, which may reflect both true epidemiological shifts and differences in diagnostic capacity (Fernandes et al., 2022; Hussen et al., 2024)

The pathogenesis of VVC is multifactorial, involving a disruption of the delicate vaginal ecosystem. A healthy vaginal microenvironment, dominated by lactobacilli, maintains a protective low pH and competitive exclusion. Dysbiosis, characterized by a decline in lactobacilli, facilitates *Candida* colonization and overgrowth (Chee et al., 2020). Host factors further modulate susceptibility; conditions such as pregnancy, diabetes, antibiotic use, immunosuppression, and certain contraceptive practices can alter local immunity and microbial balance, increasing infection risk (MacAlpine and Lionakis, 2024).

Clinically, VVC presents with pruritus, abnormal discharge, and erythema—symptoms that are nonspecific and overlap with other forms of vaginitis (Marot-Leblond et al., 2009) Diagnosis has traditionally relied on clinical evaluation supplemented by microscopy and culture. However, microscopy lacks sensitivity, while culture, though considered the gold standard for species identification, requires days for results, delaying targeted treatment (Mendonca et al., 2022). Advanced molecular diagnostics, including PCR and Matrix-assisted laser desorption/ionization time-of-flight mass spectrometry (MALDI-TOF MS), offer rapid, precise species identification and are crucial for detecting mixed infections and NAC species (Arastehfar et al., 2019). Despite these advances, distinguishing pathogenic infection from asymptomatic colonization remains a diagnostic nuance.

Growing evidence directly links antifungal exposure in VVC to resistance selection. Fluconazole use has been shown to selectively favor *N. glabratus* emergence over *C. albicans* (Nakamura-Vasconcelos et al., 2017), with clinical isolates demonstrating 100% fluconazole resistance in some cohorts (Dunaiski et al., 2024). Notably, topical azole use may also contribute, as NAC isolates exhibit substantially higher MICs to imidazoles than *C. albicans* (Choukri et al., 2014). Clinical resistance rates can be alarmingly high. For example, a 2025 study found that 54.8% of *C. albicans* isolates were resistant to clotrimazole and 53.4% to miconazole (Zaman et al., 2025). Furthermore, a 2007 study reported only 63% susceptibility to miconazole among vaginal yeast isolates, linking reduced susceptibility to frequent prior use of the drug (Gross et al., 2007). The widespread over-the-counter availability of topical azoles has raised concerns about their role in driving resistance (Gamarra et al., 2014). These findings underscore that both systemic and topical antifungal use in VVC contributes to the rising NAC resistance crisis.

Resistance rates to fluconazole can be substantial, driven by mechanisms including efflux pump overexpression, target-site mutations, altered expression of sterol biosynthesis genes, and biofilm formation (Lee et al., 2023). These mechanisms contribute to treatment failures and recurrence, complicating clinical management.

In response to these challenges, the therapeutic landscape is evolving. Novel antifungal agents like ibrexafungerp, an FDA-approved glucan synthase inhibitor, show promise against azole-resistant strains (Wiederhold et al., 2021). Research is also exploring adjunctive strategies: natural compounds (e.g., p-coumaric acid, celastrol) that inhibit biofilms or potentiate azoles (Yehia et al., 2025); nanotechnology for enhanced drug delivery (Dos Santos, Ramos et al., 2018; Pandey et al., 2020); and probiotic therapies aimed at restoring a protective microbiota (Liu et al., 2023; Zuniga Vinueza, 2024). However, the translation of these advances into practice is hindered by the lack of standardized susceptibility testing for NAC species and limited access to rapid, point-of-care diagnostics.

In summary, VVC management is at a critical juncture, shaped by changing pathogen profiles, rising antifungal resistance, and the limitations of empirical care. Advancing beyond this impasse requires an integrated approach that combines precision diagnostics, mechanism-informed therapeutics, and strategies to restore vaginal health. This review synthesizes current knowledge on the epidemiology, diagnostics, therapeutic innovations, and resistance mechanisms in VVC, aiming to provide a cohesive framework for guiding future research and improving clinical outcomes.

## 2 Literature search strategy

This narrative review synthesized peer-reviewed literature on VVC published between January 2000 and March 2026. A systematic search of PubMed/MEDLINE, Web of Science, Scopus, and Google Scholar was conducted using combinations of the following keywords and MeSH terms: “vulvovaginal candidiasis,” “VVC,” “*Candida albicans*,” “non-albicans *Candida*,” “*Nakaseomyces glabratus*,” “antifungal resistance,” “azole resistance,” “biofilm,” “diagnosis,” “PCR,” “MALDI-TOF,” “treatment,” “fluconazole,” “ibrexafungerp,” “oteseconazole” “probiotics,” and “microbiome.” Boolean operators (AND, OR) were used to refine searches (e.g., “vulvovaginal candidiasis AND (azole resistance OR biofilm)”).

Inclusion criteria: (1) original research articles, systematic reviews, meta-analyses, and clinical practice guidelines; (2) studies reporting on VVC epidemiology, diagnostics, treatment, or resistance mechanisms; (3) English language publications. Exclusion criteria: (1) case reports (except those describing novel resistance mechanisms or emerging species); (2) non-English articles; (3) studies focused exclusively on invasive candidiasis without vaginal relevance; (4) conference abstracts without full-text availability.

Two authors (W.Z. and L.G.) independently screened titles and abstracts, followed by full-text review. The final reference list prioritized high-quality evidence from the past decade while retaining seminal older studies (e.g., Sobel, 2007; Foxman et al., 2000) for historical context. Table 1 provides a categorized overview of included studies.

**TABLE 1:** Summary of included studies by topic area.

Topic area	Number of studies	Key findings summary	Representative citations
Epidemiology and risk factors	∼25	70–75% lifetime prevalence; 5–9% RVVC; NAC prevalence 10–50% varying by region	(Foxman et al., 2000; Sobel, 2007; Hussen et al., 2024; Fernandes et al., 2022)
Diagnostics (traditional)	∼10	Microscopy sensitivity 48.5–70%; culture gold standard but 24–72 h	(Danby et al., 2021; Farr et al., 2021; Nyirjesy et al., 2022)
Diagnostics (molecular)	∼12	PCR and MALDI-TOF MS offer rapid, sensitive species identification	(Arastehfar et al., 2019; Akinosoglou et al., 2024b; Zhu et al., 2025)
Antifungal susceptibility testing	∼8	pH-dependent activity; need for vagina-specific breakpoints	(Liu et al., 2011; Danby et al., 2012; Sobel et al., 2021; Sobel and Akins, 2022)
Azole therapy and resistance	∼20	Rising resistance (up to 69% in some isolates); mechanisms: ERG11 mutations, efflux pumps, UPC2 mutations	(Sobel, 2023; Lee et al., 2023; Flowers et al., 2015; Dunkel et al., 2008b)

## 3 Epidemiological characteristics and pathogen spectrum evolution

### 3.1 Epidemiology and risk factors

VVC is a widespread fungal infection among women of reproductive age, with prevalence rates exhibiting considerable geographical variation. Globally, 75% of women will experience VVC at least once in their lives, and up to 9% go on to develop RVVC (Foxman et al., 2000; Sobel, 2007; Omosa-Manyonyi et al., 2025). Research in Ethiopia found a prevalence of 26.8% among pregnant women attending antenatal care (Hussen et al., 2024), while in Portugal, the incidence among women presenting with vulvovaginitis symptoms was as high as 74.4% (Fernandes et al., 2022). However, several factors contribute to this notably high figure. First, the Portuguese study enrolled exclusively symptomatic women (presenting with vulvovaginitis), whereas many European prevalence studies include both symptomatic and asymptomatic screening populations, yielding lower overall prevalence estimates. Second, the study employed molecular techniques (PCR) rather than conventional culture, which detects lower fungal burdens and mixed infections more sensitively. Third, the species distribution in Portugal showed an unusually high proportion of *N. glabratus* (27% of isolates), which is more likely to cause symptomatic infection. Fourth, regional epidemiological patterns suggest that non-albicans *Candida* species, particularly *N. glabratus*, are more prevalent in Southern Europe compared to Northern Europe. Therefore, the 74.4% figure represents the proportion of *Candida*-positive cases among symptomatic women with PCR-based testing, not the general population prevalence of VVC in Portugal. In the United States, VVC is the second most prevalent cause of vaginal infections, impacting 70–75% of women at some point in their lives and contributing to approximately 1.4 million outpatient visits annually (Sobel, 2007; Benedict et al., 2019).

A multitude of risk factors contribute to VVC susceptibility. Pregnancy, particularly in the third trimester, is a well-documented predisposing condition due to estrogen-driven physiological changes that promote fungal adherence and growth (Disha and Haque, 2022). The use of broad-spectrum antibiotics, which disrupt the protective vaginal microbiota, and hormonal contraceptives are also strongly associated with increased risk (Sobel and Vempati, 2024). Metabolic disorders such as diabetes mellitus and immunosuppressive conditions including HIV infection significantly elevate susceptibility by impairing local and systemic host defenses (Foessleitner et al., 2021; Mohammed et al., 2021). A history of previous VVC episodes is a key predictor for recurrence, highlighting the persistent nature of the disease for some individuals (Geer et al., 2025). Beyond biological factors, behavioral and socioeconomic determinants—such as hygiene practices, the use of occlusive undergarments or panty liners, and limited access to healthcare—have been linked to infection risk, illustrating the complex interplay of host, pathogen, and environmental factors (Eskezia et al., 2025).

In summary, VVC presents a substantial and variable global health challenge. The convergence of biological, behavioral, and socioeconomic risk factors calls for a multifaceted approach to prevention and management, integrating targeted clinical care with broader public health education.

### 3.2 Changes in pathogen composition and the rise of Non-*albicans Candida* species

*C. albicans* remains the most prevalent pathogen in VVC, although its reported proportion varies substantially. Culture-based estimates typically range from 85 to 95% (Bhosale et al., 2025), whereas molecular methods detect higher rates of NAC due to improved sensitivity (Arastehfar et al., 2019). Prevalence also differs by population—symptomatic women show higher *C. albicans* dominance than asymptomatic carriers (Fernandes et al., 2022)—and by geography, with NAC species accounting for up to 30–50% of cases in Africa, the Middle East, and Asia (Okungbowa et al., 2003; Amouri et al., 2011).

However, a significant epidemiological shift is underway, marked by a rising proportion of infections attributed to non-*albicans Candida* (NAC) species (Table 2). According to epidemiological and distribution studies conducted on cohorts in the United States, Europe, and Australia, among NAC infections, *N. glabratus* exhibits the highest frequency, accounting for approximately 10–20% of cases, followed by *C. parapsilosis*, *C. tropicalis*, *P. kudriavzevii*, and *C. africana* (Linhares et al., 2001; Okungbowa et al., 2003; Cetin et al., 2007). Species distribution and incidence vary considerably by geographic region and study population, with a marked increase in NAC species among VVC patients. This trend is particularly evident in Tunisia, Nigeria, Middle Eastern countries, and Asia, where *N. glabratus* is the most frequently isolated NAC, ranging from 30 to 50% of cases (Okungbowa et al., 2003; Cetin et al., 2007; Amouri et al., 2011; Sobel, 2016). Moreover, NAC species are considered more likely to promote recurrent VVC infections (De Vos et al., 2005; Cetin et al., 2007), potentially due to their reduced susceptibility to standard azole therapy.

**TABLE 2:** Regional prevalence of non-albicans *Candida* species*.

Region/Country	Most common NAC species	Prevalence rate (among VVC cases)	Key methodological note
USA/Europe	*N. glabratus*	10–20%	Culture-based studies; may underestimate mixed infections
Tunisia/Nigeria	*N. glabratus*	30–50%	PCR/MALDI-TOF studies show higher detection rates
Asia (China, India)	*N. glabratus C. tropicalis*	15–35%	Regional variation; increasing *C. tropicalis* prevalence noted
Middle East	*C. parapsilosis*	10–25%	Higher prevalence in younger populations; requires species-level identification

*Prevalence varies significantly by diagnostic method (culture vs. molecular) and population (symptomatic vs. asymptomatic).

This evolving pathogen landscape has profound implications for clinical practice. Accurate species-level identification, facilitated by advanced methods such as MALDI-TOF MS or PCR-based assays, is now essential for guiding therapy (Zhu et al., 2025). Such precision enables tailored antifungal selection, which is critical given the variable drug susceptibility patterns among *Candida* species. The growing prevalence of azole-resistant NAC strains highlights the urgent need for routine antifungal susceptibility testing and reinforces the importance of antifungal stewardship to preserve existing drugs and guide the appropriate use of alternative agents.

These epidemiological shifts underscore the need for precise diagnostic tools, yet the complexity of the vaginal microenvironment—including its acidic pH, dynamic microbiota, and host immune responses—complicates accurate pathogen detection and interpretation.

### 3.3 Risk factors for recurrent vulvovaginal candidiasis

RVVC affects approximately 5–9% of women of reproductive age. While some affected women have no identifiable predisposing conditions, accumulating evidence has identified several contributing factors.

Host genetic factors have emerged as key determinants of RVVC susceptibility. Jaeger et al. (2013) reported that polymorphisms in Toll-like receptors (TLRs), particularly TLR2 Pro631His, may influence RVVC susceptibility by impairing vaginal mucosal antifungal immunity (Jaeger et al., 2013). Subsequent systematic reviews have confirmed the association between TLR2/TLR4 polymorphisms and RVVC (Routsias et al., 2025), with additional evidence identifying TLR4 Asp299Gly and Thr399Ile as risk variants (Rosentul et al., 2014).

Hormonal factors play a significant role. Pregnancy, particularly the third trimester, is a well-documented predisposing condition due to estrogen-driven physiological changes that promote fungal adherence and growth (Disha and Haque, 2022). High-estrogen states, including use of oral contraceptives and hormone replacement therapy, have been associated with increased VVC risk (Sobel and Vempati, 2024).

Metabolic disorders, particularly uncontrolled diabetes mellitus, significantly elevate RVVC risk by increasing vaginal glucose concentrations, which promote *Candida* growth (Mohammed et al., 2021). Immunosuppressive conditions, including HIV infection and long-term corticosteroid use, impair local host defenses and increase susceptibility (Foessleitner et al., 2021).

Antibiotic exposure within the preceding 3 months disrupts the protective vaginal microbiota, facilitating Candida overgrowth and increasing recurrence risk (Sobel and Vempati, 2024). Infection with non-albicans *Candida* species, particularly *N. glabratus*, is associated with higher recurrence rates due to intrinsic or acquired azole resistance (Makanjuola et al., 2018). Azole-resistant strains and biofilm formation further contribute to treatment failure and persistence (Fan et al., 2022; Lee et al., 2023).

Lifestyle and local factors have also been implicated. Use of non-cotton underwear, vaginal douching, local irritants (soaps, shampoos), over-the-counter antifungal overuse, and poor treatment compliance have been associated with increased RVVC risk (Fernandes et al., 2022).

Understanding these multifaceted risk factors is essential for identifying high-risk individuals and developing personalized prevention strategies.

### 3.4 Why is *C. albicans* more predominant in symptomatic patients than in asymptomatic carriers?

Epidemiological studies consistently report a higher proportion of *C. albicans* among symptomatic VVC patients compared with asymptomatic carriers. The literature provides several complementary explanations for this observation.

#### 3.4.1 Yeast-to-hypha morphological switch

In its yeast form, *C. albicans* is tolerated by the vaginal epithelium and asymptomatically colonizes the vaginal mucosa. Under morphogenesis-inducing conditions—including estrogen elevation, increased vaginal pH, and microbiome disruption—it switches to the invasive hyphal form, co-regulating the expression of genes encoding virulence factors such as secreted aspartyl proteases (Saps) and the pore-forming toxin candidalysin, which enable tissue invasion and epithelial damage. This morphology switching, coupled with increasing fungal burden, can overcome the host’s tolerance threshold and trigger an intense inflammatory response (Ardizzoni et al., 2021).

#### 3.4.2 Activation of host inflammatory response

A 2017 study directly compared VVC patients (symptomatic), asymptomatic carriers, and non-colonized women, finding that overexpression of NLRP3 and caspase-1 inflammasome components sharply differentiated symptomatic patients from asymptomatic carriers (Roselletti et al., 2017). Inflammasome expression was coupled with neutrophil recruitment in the vagina of VVC women and IL-1β and IL-8 production. Hyphae-associated genes (*HWP1*, *ECE1*) and the secreted aspartyl proteinase gene *SAP2* were significantly upregulated in VVC patients compared with asymptomatic carriers (Roselletti et al., 2017). Candidalysin, a peptide toxin secreted by *C. albicans* hyphae, drives epithelial damage, immune activation, and neutrophil attraction; mice challenged with candidalysin-deficient strains exhibited no differences in colonization compared with isogenic controls, but significant decreases in neutrophil recruitment, damage, and proinflammatory cytokine expression were observed (Richardson et al., 2018). Importantly, recruited neutrophils appear ineffective at reducing fungal burden and may contribute more to the symptoms associated with vaginitis than to protection against the disease.

#### 3.4.3 Differential virulence potential

A 2025 comparative study of *C. albicans* isolates from RVVC patients versus asymptomatic vaginal colonizers demonstrated that isolates from symptomatic patients exhibited significantly enhanced biofilm formation, higher expression of virulence factor genes (SAP, PL, Lip, ALS1, ALS3, HWP1), and increased antifungal resistance (Yan et al., 2025). A comprehensive 2025 review notes that asymptomatic colonization remains surprisingly understudied, but the mucosal environment shapes *C. albicans* adaptations that maintain or even increase pathogenic potential (Schille et al., 2025).

Collectively, these findings indicate that symptomatic infection arises not merely from the presence of the fungus but from a combination of morphological transition, virulence factor expression, and an exaggerated host inflammatory response that distinguishes pathogenic from commensal states.

### 3.5 Drivers of geographic variation in *Candida* species distribution

Geographic differences in *Candida* species distribution are multifactorial. Climate is a key driver: *C. tropicalis* is enriched in tropical regions, with temperature and aridity selecting for thermotolerant species, while *C. albicans* predominates in cooler areas such as North/Central Europe and the USA (Falagas et al., 2010). Antifungal use patterns also contribute significantly. Data from the ARTEMIS DISK Global Antifungal Surveillance Program showed substantial geographic variation in *N. glabratus* isolation rates, from 7.4% in Latin America to 21.1% in North America, with the highest fluconazole resistance rates observed in Poland (22%), the Czech Republic (27%), Venezuela (27%), and Greece (33%). Regions with higher azole consumption, particularly over-the-counter availability, tend to report higher NAC prevalence (Pfaller et al., 2010). Urban versus rural residence affects distribution as well: a Tanzanian study reported NAC prevalence of 57% in rural women versus 32% in urban women, with untreated water use and drying clothes in bushes significantly associated with NAC presence (Namkinga et al., 2025). Ethnicity and host genetics play a role: a Chinese study found *C. albicans* accounted for 77.1% of isolates from Han women versus 89.6% from Tibetan women living in the same geographic region (Wei et al., 2010). Finally, healthcare infrastructure influences reported distribution, as regions with access to molecular diagnostics detect higher NAC rates than those relying solely on culture (Akinosoglou et al., 2024b). These factors collectively shape regional epidemiological patterns and underscore the need for localized surveillance to guide empirical therapy.

## 4 Advances and challenges in diagnostic technologies

### 4.1 Traditional microbiological diagnostic methods

Traditional diagnosis of VVC relies on direct microscopy and culture-based techniques. Wet mount microscopy and Gram staining serve as accessible, low-cost initial screens, providing rapid results that can immediately guide empirical therapy. However, their sensitivity is operator-dependent and often inadequate, with reported values ranging from 48.5 to 70% for detecting *Candida* in symptomatic women (Danby et al., 2021; Sivagamasundari et al., 2024). This limitation is particularly pronounced when fungal burden is low or in mixed infections, leading to a significant rate of false negatives and underscoring the risk of underdiagnosis if microscopy is used as the sole diagnostic method (Farr et al., 2021). Culture remains the diagnostic gold standard, allowing for species identification and antifungal susceptibility testing. The use of selective media, such as Sabouraud Dextrose Agar (SDA), and differential chromogenic agars (e.g., CHROMagar Candida) enables the presumptive differentiation of *Candida albicans* from key non-*albicans* species based on colony color and morphology. While indispensable, culture has inherent limitations: it requires 24–72 h for growth, delaying definitive diagnosis, and its yield can be reduced by prior antifungal exposure or suboptimal sample transport (Nyirjesy et al., 2022).

Biochemical identification systems (e.g., API 20C AUX) offer additional specificity but are time-consuming, require isolated colonies, and may fail to identify rare or emerging species (Arastehfar et al., 2019). Overall, traditional methods provide a necessary foundation, particularly in resource-constrained settings, but their constraints in speed, sensitivity, and scalability underscore the need for more advanced diagnostic solutions in modern clinical practice.

### 4.2 Molecular and rapid diagnostic techniques

Molecular diagnostics have transformed the laboratory identification of *Candida* by delivering high sensitivity, specificity, and rapid turnaround. Techniques such as PCR-RFLP and real-time PCR enable direct detection and differentiation of species from clinical specimens, often within hours, bypassing the need for culture (Eghtedar Nejad et al., 2020; Jabrodini et al., 2024). This is crucial for detecting mixed infections and low-abundance pathogens that microscopy or culture may miss. MALDI-TOF MS has revolutionized identification from cultured isolates, providing species-level results in minutes with high accuracy, thereby streamlining laboratory workflows (Zhu et al., 2025).

These technologies offer profound clinical advantages. They facilitate the precise etiological diagnosis required for managing NAC infections and RVVC, where identifying the infecting strain (e.g., to distinguish relapse from reinfection) can inform therapeutic strategy. The future direction lies in translating this precision to the point of care. Emerging rapid diagnostic tests, such as nucleic acid amplification tests (NAATs) (Danby et al., 2021) and antigen-detection biosensors (Lorenzo-Villegas et al., 2023), aim to provide accurate, species-specific results during a clinical visit. For instance, nanoparticle-based colorimetric assays have shown promise for detecting *C. albicans* directly from samples in under an hour, offering a potential paradigm shift for settings lacking advanced laboratory infrastructure (Clack et al., 2024; Suaifan et al., 2024; Qiu et al., 2026).

**FIGURE 1:**
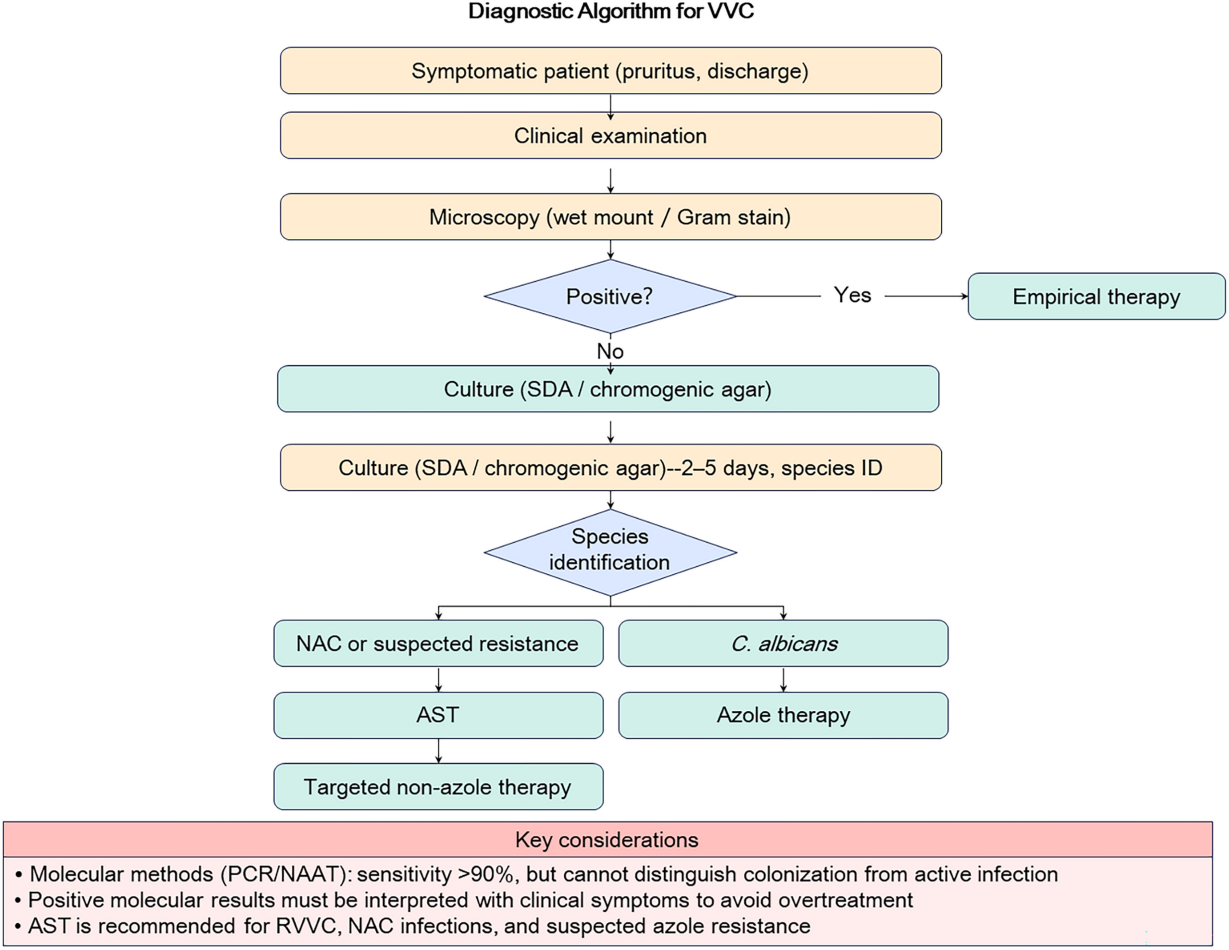
Diagnostic algorithm for vulvovaginal candidiasis. The algorithm illustrates a stepwise approach to diagnosis, integrating clinical presentation with traditional methods (microscopy, culture) and molecular techniques. A key interpretative pitfall is highlighted: molecular methods detect nucleic acids and cannot distinguish colonization from active infection. AST, antifungal susceptibility testing; NAC, non-albicans Candida; RVVC, recurrent vulvovaginal candidiasis.

Despite their advantages, the widespread adoption of molecular diagnostics faces significant barriers. High costs per test and the requirement for specialized laboratory infrastructure and trained personnel limit accessibility in resource-limited settings (Akinosoglou et al., 2024b). Perhaps more critically, a major interpretative pitfall is that molecular methods detect nucleic acids, which may represent either active infection or asymptomatic colonization. This distinction is crucial to avoid over-treatment (Deckers et al., 2025). Therefore, positive molecular results must always be interpreted in the context of clinical symptoms and microscopic findings, underscoring the continued importance of a combined clinical and laboratory approach (Figure 1).

### 4.3 Standardization and controversies in antifungal susceptibility testing

Antifungal susceptibility testing (AST) is a critical component of managing VVC, especially in recurrent or refractory cases. The standardized broth microdilution method (CLSI M27 (CLSI, 2017) or EUCAST (2021)) provides essential reference data for interpreting resistance. However, translating these standards to the clinical context of VVC presents specific challenges and controversies.

A central debate revolves around the environmental conditions for testing. Standard AST is performed at neutral pH (∼7.0), whereas the vaginal environment is acidic—typically pH ∼4.5 under healthy conditions, dropping to a range of 3.8–4.5 during candidiasis (Sobel et al., 2021). This discrepancy is not trivial, as the activity of azole antifungals—the cornerstone of VVC therapy—is known to be pH-dependent. Evidence suggests that testing at vaginal pH may better correlate with clinical outcomes by more accurately revealing azole resistance profiles relevant to the site of infection (Liu et al., 2011). This has led to calls for adjunctive testing under acidic conditions to improve the clinical predictive value of AST for vaginal isolates. However, the clinical relevance of this pH adjustment remains controversial. While some studies demonstrate that testing at acidic conditions reveals clinically relevant resistance that correlates with treatment failure (Danby et al., 2012), others argue that the short exposure time of topical azoles in the vagina or the buffering capacity of the formulation may mitigate the impact of pH (Liu et al., 2011). Moreover, the lack of standardized, validated methodology for acidic pH testing prevents its integration into routine clinical laboratory practice. This discrepancy highlights an urgent need for multicenter studies correlating pH-adjusted MICs with clinical outcomes to establish definitive, vagina-specific breakpoints (Sobel and Akins, 2022).

Furthermore, interpretive breakpoints for susceptibility are primarily derived from data on systemic candidiasis. The pharmacokinetics and pharmacodynamics of antifungals in the vaginal compartment differ significantly, raising questions about the direct applicability of these breakpoints to localized VVC. Establishing clinically validated, vagina-specific breakpoints, particularly for azoles, remains an urgent unmet need to guide therapy and define resistance in this setting.

The problem is compounded by the rising prevalence of non-*albicans* species with intrinsic or acquired resistance (e.g., *N. glabratus*, *P. kudriavzevii*) and the contribution of biofilm growth, which is poorly assessed by standard planktonic AST. In summary, while standardized AST provides a vital framework, its optimization for VVC requires addressing site-specific physiological conditions, developing relevant clinical breakpoints, and considering novel methods to account for biofilm-associated tolerance.

## 5 Current antifungal treatment strategies and their limitations

### 5.1 Azoles: challenges in first-line treatment

Azole antifungals, including topical clotrimazole/miconazole and oral fluconazole, remain the first-line therapy for acute VVC due to efficacy and ease of use (Satora et al., 2023). They inhibit lanosterol 14α-demethylase (encoded by *ERG11*), disrupting fungal membrane synthesis. However, their clinical utility is increasingly threatened by rising resistance. Fluconazole resistance rates among *Candida* VVC isolates have been reported as high as ∼69%, with significant cross-resistance observed for other azoles. This crisis is driven by two key factors: the emergence of non-*albicans Candida* (NAC) species and the evolution of resistance in *C. albicans* (Zaman et al., 2022; Sobel, 2023; Akinosoglou et al., 2024a).

NAC species, particularly *N. glabratus* and intrinsically resistant *P. kudriavzevii*, frequently exhibit reduced azole susceptibility, making empirical therapy risky (Pfaller et al., 2008; Castanheira et al., 2022; Zheng et al., 2024; Jain et al., 2026). In *C. albicans*, resistance is mediated primarily by *ERG11* mutations, the overexpression of efflux pumps (e.g., *CDR1*, *MDR1*), and gain-of-function mutations in transcriptional regulators such as *UPC2* that lead to constitutive activation of the ergosterol biosynthesis pathway. These mechanisms have been directly linked to high MIC values and clinical treatment failure (Lee et al., 2023).

The clinical consequences are clear: azole resistance is a major contributor to treatment failure and recurrent VVC (RVVC). Compounding this, standard antifungal susceptibility testing performed at neutral pH may underestimate resistance relevant to the acidic vaginal environment. Consequently, while azoles remain a cornerstone, their empirical use is no longer tenable in many cases. This necessitates a shift toward diagnostics-driven therapy, where species identification and susceptibility testing guide treatment selection, and underscores the urgent need for accessible, non-azole alternatives.

### 5.2 Polyenes and echinocandins

When azoles fail, polyenes and echinocandins provide essential alternative mechanisms of action.

#### 5.2.1 Polyenes (e.g., nystatin, amphotericin B)

These drugs bind to ergosterol, disrupting the fungal cell membrane (Carolus et al., 2020). Topical nystatin retains near-universal in vitro activity against both *C. albicans* and NAC species, making it a valuable, safe alternative for azole-resistant or intolerant cases, including during pregnancy. Its role is limited to topical use. Systemic amphotericin B, while potent against resistant strains, is reserved for severe, life-threatening infections due to significant toxicity (e.g., nephrotoxicity) and the need for intravenous administration (Chandrasekar, 2011).

#### 5.2.2 Echinocandins (e.g., caspofungin)

This class inhibits β-1,3-D-glucan synthase, a key enzyme for cell wall synthesis. Echinocandins are potent and fungicidal against most *Candida* species, including azole-resistant strains, and are first-line for invasive candidiasis. However, their role in VVC management is severely constrained. Despite *in vitro* potency, echinocandins lack a topical formulation, and intravenous administration is impractical for a non-life-threatening mucosal infection. Therefore, they offer genuine added value only in exceptionally rare, severe cases of refractory VVC where systemic infection is a concern or where all other treatment options have been exhausted. For routine azole-resistant VVC, echinocandins are not a practical first-line alternative (Pappas et al., 2016).

In summary, while polyenes and echinocandins are crucial for resistant or severe infections, their practical application in routine VVC is hampered by formulation and toxicity issues. Topical nystatin stands out as the most practical alternative for localized, azole-resistant cases.

### 5.3 Management of recurrent and refractory VVC

RVVC represents a therapeutic challenge often intertwined with antifungal resistance. The traditional strategy of induction followed by long-term azole maintenance is increasingly ineffective against resistant strains selected by repeated drug exposure (Arastehfar et al., 2021). Management must therefore be precise and multifaceted:

1. *Precise diagnosis*: Species identification and antifungal susceptibility testing are imperative to guide therapy, moving beyond empirical azole re-challenge (Neal and Martens, 2022).

*Alternative pharmacotherapy:* For azole-resistant cases, vaginal boric acid remains a highly effective and low-cost option, particularly against NAC species such as *P. kudriavzevii* (Singh et al., 2002). Beyond these traditional alternatives, novel oral agents have emerged as promising systemic options. Ibrexafungerp, a first-in-class triterpenoid antifungal, inhibits glucan synthase via a unique mechanism and has demonstrated efficacy against azole-resistant Candida isolates in clinical trials, positioning it as a valuable tool for RVVC (Sobel et al., 2021). Similarly, oteseconazole, a highly selective fungal CYP51 inhibitor, has recently gained FDA approval specifically for reducing the incidence of RVVC in women with a history of recurrent infections. Unlike traditional azoles, oteseconazole exhibits a longer half-life and is administered as a maintenance regimen following initial antifungal therapy, offering a targeted option for sustained prevention (Lanier and Melton, 2024). Together, these novel agents represent a significant advancement beyond the limitations of chronic azole suppression. Meanwhile, ongoing research into combination strategies—such as azoles paired with efflux pump inhibitors like farnesol—aims to overcome specific resistance mechanisms (Dekkerova et al., 2022).

For azole-resistant cases, vaginal boric acid is a highly effective, low-cost option, especially against NAC species like *P. kudriavzevii*. Novel oral agents like ibrexafungerp (a triterpenoid antifungal) show promise against resistant isolates and offer a much-needed systemic alternative. Research into combination therapies (e.g., azoles with efflux pump inhibitors like farnesol) aims to overcome specific resistance mechanisms. In this context, ibrexafungerp, a first-in-class triterpenoid antifungal, has emerged as a promising systemic alternative. Its unique mechanism of inhibiting glucan synthase, combined with oral bioavailability and demonstrated efficacy against azole-resistant Candida in clinical trials, positions it as a valuable tool for RVVC. Similarly, oteseconazole, a highly selective fungal CYP51 inhibitor, has recently gained FDA approval specifically for reducing the incidence of RVVC, offering a targeted option for maintenance therapy. These novel agents represent a significant advancement beyond the limitations of chronic azole suppression. Both agents have received FDA approval and are now available in clinical practice—ibrexafungerp for acute VVC and RVVC reduction, and oteseconazole specifically for RVVC in women without reproductive potential—representing a major advancement beyond chronic azole suppression.

2. *Adjunctive and non-pharmacological strategies*: Given the role of dysbiosis in recurrence, strategies to restore a protective microbiota are key. Specific probiotic strains (e.g., *Lactobacillus*) can inhibit *Candida* adhesion and biofilm formation (Matsubara et al., 2016; Takano et al., 2023), and innovative delivery systems (e.g., microencapsulation) are being developed to enhance their efficacy and viability in the vagina (Kristina Enggi et al., 2023).

Ultimately, successful RVVC management requires an integrated approach: leveraging diagnostics to choose the right drug, incorporating non-azole alternatives when needed, and addressing the underlying vaginal ecologic imbalance to prevent relapse and reduce dependence on antifungal suppression.

### 5.4 Clinical translation of emerging therapies

Several novel antifungal agents discussed in this review have already entered clinical practice for VVC and RVVC management. Ibrexafungerp (Brexafemme^®^), a first-in-class triterpenoid glucan synthase inhibitor, received FDA approval in June 2021 for the treatment of VVC, followed by approval in December 2022 for reduction of RVVC incidence based on the phase III CANDLE trial, which demonstrated that 65.4% of ibrexafungerp-treated patients remained recurrence-free at 24 weeks compared to 53.1% in the placebo group (*p* = 0.02) (Sobel et al., 2021; Wiederhold et al., 2021). Oteseconazole (Vivjoa^®^), a highly selective fungal CYP51 inhibitor, was approved by the FDA in April 2022 as the first and only therapy specifically indicated for RVVC in women with no reproductive potential. Phase III VIOLET trials showed that 96% of oteseconazole-treated patients remained infection-free at 48 weeks (vs. 61% for placebo, *p* < 0.001), with extended data demonstrating 98% recurrence-free at 96 weeks (Lanier and Melton, 2024). Boric acid, while not FDA-approved specifically for VVC, is recommended by CDC guidelines for RVVC caused by non-albicans *Candida* species, particularly *N. glabratus*, and is widely used in clinical practice (Singh et al., 2002).

In addition to approved therapies, multiple clinical trials are actively evaluating emerging approaches. A phase III trial of ibrexafungerp for complex VVC with prior fluconazole failure has been completed (NCT05399641). A phase III randomized, double-blind, placebo-controlled trial of boric acid 600 mg vaginal suppository for 7 or 14 days is currently recruiting (NCT07109869). A real-world observational study of oteseconazole in 3000 Chinese VVC patients is ongoing (NCT07044947). A phase II trial of WXSH0102, a novel oral antifungal, has been completed in China (NCT06771063). Finally, a completed phase IV trial evaluated probiotic (Lactobacillus) supplementation combined with clotrimazole for RVVC prevention (NCT04699240), supporting the adjunctive role of microbiome modulation.

These clinical developments underscore a paradigm shift from empirical azole-based regimens toward evidence-based, mechanism-informed therapeutic strategies (Box 1).

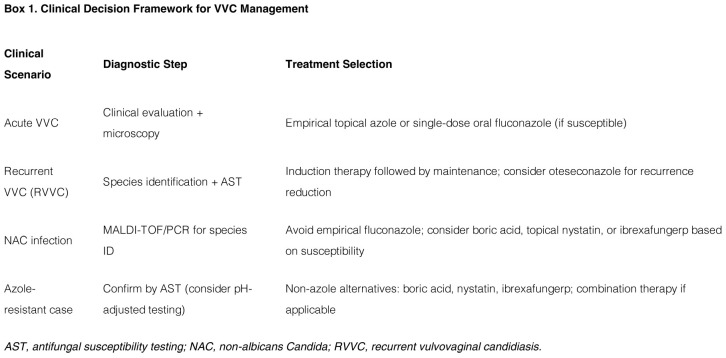

## 6 Emerging therapeutic strategies and research directions

### 6.1 Novel antifungal drugs and dosage form development

The rising challenge of antifungal resistance has spurred significant innovation in drug discovery and delivery for VVC. A key advance is the development of next-generation triazoles with improved efficacy against resistant strains. For instance, ravuconazole has demonstrated potent *in vitro* activity against multidrug-resistant species like *C. auris* and showed a remarkably low resistance rate (0.19%) in a large collection of vaginal *Candida* isolates, positioning it as a promising oral candidate for refractory infections (Dong et al., 2021).

Concurrently, advanced local delivery systems are being engineered to enhance drug bioavailability, residence time, and patient compliance. These innovations include:

- *Sustained-release formulations:* Hydrogels and mucoadhesive systems (e.g., those based on chitosan, poloxamer, or cyclodextrin) provide controlled release of antifungals like terbinafine, improving local drug persistence (Arpa et al., 2020; Samra et al., 2025).
- *Nanotechnology platforms*: Nanocapsules, nanomicelles, and nanofibers can encapsulate both traditional antifungals (e.g., clotrimazole; Helal et al., 2025) and natural compounds (e.g., *Cordyceps* polysaccharides; Lu et al., 2023, myrtle extracts, Bellu et al., 2022), enhancing stability, penetration, and targeted delivery to the vaginal mucosa.
- *Probiotic delivery systems*: Innovative platforms like probiotic-loaded microcapsules incorporated into soluble microneedles are designed to improve the survival and colonization of beneficial bacteria (e.g., *Lactobacillus*) directly at the infection site (Kristina Enggi et al., 2023).

These strategies aim to overcome key limitations of current therapies by maximizing local therapeutic effect while minimizing systemic exposure and resistance selection.

### 6.2 Application of antimicrobial peptides and nanomaterials

Antimicrobial peptides and engineered nanomaterials offer novel, mechanism-based approaches to combat drug-resistant and biofilm-associated VVC.

- *Antimicrobial peptides*: Peptides such as Epinecidin-1 and its derivatives exhibit broad-spectrum fungicidal activity by disrupting cell membranes, inducing oxidative stress, and critically, disrupting biofilms. They target key biofilm components and virulence factors, showing efficacy against resistant species like *P. kudriavzevii* in preclinical models (Jeyarajan et al., 2024).
- *Metal-based nanomaterials*: Nanoparticles, particularly those of copper (CuO, CuS), exhibit potent, concentration-dependent antifungal and antibiofilm activity. Their mechanism involves generating reactive oxygen species and damaging fungal cell structures. Some formulations show enhanced activity when conjugated with targeting molecules (e.g., lectins) or combined with conventional antifungals, suggesting potential for synergistic therapy (Munoz-Escobar and Reyes-Lopez, 2020; Carmo et al., 2023).

These agents are particularly valuable for their ability to target multiple pathways and overcome common resistance mechanisms like efflux pumps and biofilm-mediated tolerance. However, their translation to clinical use requires further investigation into long-term safety, pharmacokinetics, and formulation for vaginal application.

### 6.3 Probiotics and microbiome modulation

Modulating the vaginal microbiome represents a paradigm-shifting, non-antifungal strategy for treating and preventing VVC, especially recurrence. The therapeutic rationale is to restore a protective, lactobacillus-dominated ecology that competitively excludes *Candida*.

- *Mechanisms of action*: Protective lactobacilli (e.g., *L. crispatus*, *L. rhamnosus*) work through multiple mechanisms: producing lactic acid to maintain a low pH; secreting antimicrobial substances like biosurfactants and bacteriocins; competitively blocking epithelial adhesion sites; and modulating local immune responses to reduce pathological inflammation (Vazquez-Munoz and Dongari-Bagtzoglou, 2021).
- *Therapeutic evidence*: Probiotic formulations, both as live bacteria and their cell-free supernatants, have demonstrated efficacy in preclinical studies. They inhibit *Candida* hyphal transformation, disrupt biofilms, and downregulate virulence genes. Combinations with certain compounds (e.g., sulfur derivatives) can potentiate these effects (Dausset et al., 2020; Poon and Hui, 2023; Takano et al., 2023).
- *Delivery innovation*: Research is focused on improving probiotic viability and local delivery through microencapsulation and other stabilization technologies to ensure sufficient numbers of beneficial bacteria reach and colonize the vaginal epithelium (Kristina Enggi et al., 2023; Liu et al., 2023). This approach aims to address the root cause of susceptibility in many women, offering a sustainable strategy to reduce recurrence and dependence on antifungal drugs.

## 7 Molecular mechanisms of drug resistance

Antifungal resistance in *Candida* species is mediated by diverse molecular mechanisms that vary by drug class. Azoles, the cornerstone of VVC therapy, are most extensively studied; resistance primarily arises from *ERG11* mutations, efflux pump overexpression (*CDR1*, *MDR1*), and transcriptional regulator alterations (*UPC2*, *TAC1*, *MRR1*), as detailed in sections 5.1–5.3. For polyenes (e.g., amphotericin B, nystatin), resistance is uncommon but can occur via *ERG* gene mutations that alter membrane sterol composition, thereby reducing drug binding (Carolus et al., 2020). Echinocandins (e.g., caspofungin) target β-1,3-D-glucan synthase; resistance is typically mediated by hotspot mutations in *FKS1* or *FKS2*, though their use in VVC is limited by the lack of topical formulations (Perlin, 2015). Flucytosine, rarely used as monotherapy in VVC, is inactivated via mutations in *FCY1, FCY2*, or *FUR1*, leading to rapid resistance development (Vermes et al., 2000). A comprehensive summary of these resistance mechanisms is provided in Table 3.

**TABLE 3:** Summary of major antifungal resistance mechanisms in *Candida* species.

Drug class	Key resistance mechanisms	Key genes and clinical relevance
Azoles	– Target enzyme alterations – Efflux pump overexpression – Transcriptional regulator mutations	*ERG11*, CDR1, CDR2, *MDR1*, *UPC2* Clinical: Major cause of RVVC; widespread in *C. albicans* and NAC
Polyenes	– *ERG* gene mutations – Biofilm-mediated tolerance	*ERG2*, *ERG3*, *ERG6* Clinical: Resistance rare; biofilms reduce efficacy
Echinocandins	– FKS1/FKS2 hotspot mutations – Acquired resistance	*FKS1*, *FKS2* Clinical: Rare in VVC (no topical formulation)
Flucytosine	– FCY1/FCY2/FUR1 mutations – Loss of drug activation	*FCY1*, *FCY2*, *FUR1* Clinical: Rarely used in VVC; rapid resistance development
Biofilm (all classes)	– Extracellular matrix barrier – Persister cells – Efflux pump upregulation	*ALS3*, *HWP1*, EPS components Clinical: Major contributor to recurrence; requires biofilm-targeting strategies

### 7.1 Target enzyme alterations and gene mutations

Azole antifungals target lanosterol 14α-demethylase, encoded by the *ERG11* gene. Resistance arises through alterations that reduce drug binding or increase target abundance. The primary mechanisms are:

- *Point mutations*: Specific amino acid substitutions (e.g., Y132F, K143R in *C. albicans*) in the enzyme’s active site diminish azole binding affinity without compromising its catalytic function (Flowers et al., 2015).
- *Gene overexpression*: Increased *ERG11* copy number or transcriptional upregulation elevates enzyme levels, requiring higher drug concentrations to achieve inhibition (Selmecki et al., 2006; Selmecki et al., 2008).

These mechanisms often coexist, leading to high-level resistance. They are prevalent not only in *C. albicans* but also contribute to intrinsic and acquired resistance in non-*albicans* species like *N. glabratus, C. tropicalsis, C. parapsilosis*, and *C. auris* (Rybak et al., 2021; Vu and Moye-Rowley, 2022; Doorley et al., 2023; Chitharagi et al., 2025).

### 7.2 *UPC2* transcriptional regulatory mutations

Beyond mutations within the *ERG11* gene itself, alterations in its upstream regulatory factors constitute a significant mechanism of azole resistance. *UPC2*, a key transcription factor governing sterol biosynthesis, plays a central role in maintaining fungal membrane homeostasis. It activates the transcription of multiple ergosterol pathway genes, including *ERG2*, *ERG3*, and *ERG11*, by binding to sterol response elements (SREs) (Oliver et al., 2007; Vasicek et al., 2014).

Gain-of-function mutations in *UPC2* (e.g., G648D, A643T in *C. albicans*) result in its constitutive activation. This leads to persistent upregulation of ergosterol biosynthetic genes even in the presence of drug pressure or low sterol environments, thereby compensating for the fungistatic effect of azoles and conferring high-level tolerance (Dunkel et al., 2008b; Heilmann et al., 2010).

### 7.3 Overexpression of efflux pumps

The active expulsion of drugs from fungal cells via efflux pumps is a major cause of multidrug resistance. Two families are particularly significant: the ABC transporters (e.g., *CDR1*, *CDR2*) and major facilitator superfamily (MFS) pumps (e.g., *MDR1*). Clinically relevant resistance stems from the constitutive overexpression of these pumps, typically driven by gain-of-function mutations in their transcriptional regulators (*TAC1* for ABC pumps, *MRR1* for MDR1). This mechanism is frequently observed in biofilm-associated cells and recurrent isolates (Coste et al., 2006; Dunkel et al., 2008a; Schubert et al., 2011). Consequently, efflux pump inhibitors are being explored as adjunctive therapies to “resensitize” resistant strains to azoles. The development of such inhibitors, often derived from natural products, represents a promising strategy to restore the efficacy of existing antifungal drugs (Keniya et al., 2015; Yang et al., 2023).

### 7.4 Biofilm formation: a primary driver of resistance and persistence

Biofilm formation is a central mechanism enabling *Candida* species to evade antifungal treatment and establish persistent vulvovaginal infections. These structured communities, embedded within a protective extracellular matrix, are found in a significant proportion of clinical cases and confer up to a 1,000-fold increase in drug tolerance. The biofilm matrix acts as a formidable physical barrier, restricting drug penetration. Furthermore, biofilms harbor metabolically dormant “persister” cells that survive antifungal exposure and can reseed infection (LaFleur et al., 2006).

Resistance within biofilms is multifactorial: the matrix limits drug diffusion, while the enclosed cells upregulate efflux pumps and shift to a quiescent metabolic state (Table 4). Key virulence factors, such as the adhesin Als3 and hyphal wall protein Hwp1, mediate initial attachment to vaginal epithelium and are essential for robust biofilm architecture. Their expression is regulated by signaling pathways like cAMP-PKA, which controls the critical yeast-to-hyphae transition (Pereira et al., 2021; Fan et al., 2022).

**TABLE 4:** Biofilm-associated resistance pathways in *Candida* species.

Pathway/component	Key features	Molecular/structural Basis	Clinical relevance	Potential intervention
Biofilm formation	– Adhesion to vaginal epithelium – Yeast-to-hyphae transition – Secretion of extracellular polymeric substance (EPS)	– Als3, Hwp1 adhesins – cAMP-PKA signaling pathway – EPS composed of β-glucan, mannans, DNA	Found in a high proportion of recurrent VVC cases; biofilm formation correlates with persistence and recurrence	– Probiotics (*Lactobacillus* spp.) inhibit adhesion – Natural compounds (e.g., clove oil) block hyphal transition
Physical barrier (EPS matrix)	– Limits drug penetration into biofilm – Drug concentration gradient within biofilm	– EPS acts as a diffusion barrier – Binds and sequesters antifungal agents	Contributes to suboptimal drug concentrations at infection site; requires higher drug doses for efficacy	– Enzymes (e.g., β-glucanase, DNase) degrade EPS components – Novel drug delivery systems (nanoparticles)
Persister cells	– Metabolically dormant subpopulation – Survive high drug concentrations – Can reseed infection after therapy	– ATP depletion, reduced metabolic activity – Toxin-antitoxin systems (putative) – Not genetically resistant	Major cause of treatment failure and recurrence; persister cells are not detected by standard AST	– Therapies targeting dormant cells – Combination therapy to eliminate persisters – Biofilm-disrupting agents
Efflux pump upregulation	– Active drug extrusion from cells – Overexpressed in biofilm cells	– ABC transporters (Cdr1, Cdr2) – Major facilitator superfamily (Mdr1) – Biofilm-induced transcriptional changes	Contributes to multidrug resistance; reduces intracellular drug accumulation	– Efflux pump inhibitors (e.g., farnesol, natural compounds) – Non-azole alternatives (nystatin, ibrexafungerp)
Clinical consequences	– Recurrent VVC (RVVC) – Treatment failure – Need for prolonged or alternative therapy	– Multifactorial: combined effects of EPS, persisters, efflux pumps	Up to 1000-fold increase in drug tolerance compared to planktonic cells; requires biofilm-targeting strategies	– Biofilm-specific treatment protocols – Combination therapy (antifungal + biofilm disruptor) – Preventive strategies (probiotics, microbiome modulation)

This understanding directs novel therapeutic strategies. Interventions aim to prevent biofilm formation or disrupt mature structures. Natural compounds (e.g., clove oil) can inhibit genes essential for hyphal growth (Gao et al., 2025), while biosurfactants produced by protective *Lactobacillus* species interfere with *Candida* adhesion (Monti et al., 2025). Agents like chlorhexidine show superior biofilm-disrupting activity compared to fluconazole alone, highlighting the potential of combination therapies (Alvendal et al., 2020). Targeting biofilm formation and integrity is thus a crucial frontier for overcoming treatment failure in RVVC (Yan et al., 2025).

### 7.5 The host microenvironment

The vaginal ecosystem is a critical determinant of antifungal efficacy and resistance evolution. Vaginal pH (∼4.5) can directly alter the activity and protonation state of azoles; standard susceptibility testing at neutral pH may therefore underestimate clinically relevant resistance, arguing for the use of physiologically relevant conditions in laboratory assays (Liu et al., 2011; Sobel et al., 2021). Beyond pH, host immune and hormonal factors profoundly shape the susceptibility to and persistence of VVC.

The IL-17 axis plays a central role in mucosal antifungal immunity. Th17 cells and their signature cytokine IL-17 are essential for recruiting neutrophils and inducing antimicrobial peptides (such as defensins and calprotectin) at the vaginal mucosa, thereby controlling *Candida* overgrowth (MacAlpine and Lionakis, 2024). Clinical studies have demonstrated that women with RVVC often exhibit impaired Th17 responses, including reduced IL-17 production upon *Candida* stimulation, suggesting that subtle defects in this pathway contribute to recurrence susceptibility (Karjalainen et al., 2019). From a therapeutic perspective, these findings raise the possibility that immunomodulatory strategies—such as enhancing Th17 function or administering IL-17—could represent future adjunctive approaches for RVVC, though this remains an investigational frontier.

Host genetic susceptibility also contributes to VVC risk. Emerging evidence has identified polymorphisms in genes involved in antifungal immunity, particularly *CARD9* (caspase recruitment domain-containing protein 9), which is critical for downstream signaling following fungal recognition. Loss-of-function *CARD9* mutations are known to confer susceptibility to severe or recurrent candidiasis in rare cases, while common variants may modulate risk in broader populations (Omosa-Manyonyi et al., 2025). Similarly, polymorphisms in IL-22 and other cytokine genes have been associated with altered susceptibility to RVVC. Although these genetic factors are not currently used in routine clinical practice, they hold promise for identifying women at highest risk of recurrence and may eventually inform individualized prevention strategies.

Hormonal regulation, particularly estrogen, exerts a dual influence on VVC pathogenesis. Estrogen promotes *Candida* adherence to vaginal epithelial cells and enhances hyphal formation and biofilm development—key virulence traits that facilitate tissue invasion and antifungal tolerance (Goncalves et al., 2020; Disha and Haque, 2022). At the same time, estrogen modulates local immune responses, including effects on Th17 differentiation and the integrity of the epithelial barrier. These hormone-driven mechanisms help explain the peak incidence of VVC during reproductive years and the protective effect of menopause. Clinically, this hormonal influence underscores the importance of considering a patient’s hormonal status when managing recurrent infections and supports the rationale for strategies that modulate the vaginal environment, such as probiotic restoration, rather than relying solely on antifungal suppression.

In summary, the host microenvironment—encompassing pH, immune signaling, genetic predisposition, and hormonal status—interacts dynamically with Candida to determine infection outcomes. Understanding these host factors not only illuminates mechanisms of recurrence but also opens avenues for personalized prevention and novel therapeutic approaches beyond conventional antifungals.

## 8 Conclusion

VVC remains a significant clinical and public health challenge, with its management increasingly complicated by an evolving epidemiological and resistance landscape. The rising prevalence of non-*albicans Candida* species, frequently harboring intrinsic or acquired antifungal resistance, necessitates a fundamental shift away from empirical, one-size-fits-all approaches toward precision-based care.

The future of VVC management hinges on the integration of rapid, precise diagnostics with mechanism-informed therapy. Molecular diagnostic tools are essential for accurate species identification and detecting resistance markers, while antifungal susceptibility testing must be contextualized within the physiological vaginal environment to predict clinical efficacy reliably. This diagnostic precision is the critical first step in curbing the overuse and misuse of antifungals that drives resistance.

Therapeutic strategies are expanding beyond traditional azoles. While azoles remain useful for susceptible infections, the growing resistance crisis underscores the importance of alternative agents—from topical polyenes to novel systemic drugs—and the exploration of innovative approaches. These include antimicrobial peptides, nanotechnology-enhanced delivery systems, and strategies targeting biofilm disruption or virulence factors, and approaches aimed at inhibiting key resistance regulators. Concurrently, modulating the vaginal microbiome with evidence-based probiotics represents a promising avenue for preventing recurrence and restoring ecological balance.

From a translational perspective, it is noteworthy that several agents discussed in this review have already moved beyond the bench to clinical application. Ibrexafungerp and oteseconazole have received FDA approval for VVC and RVVC, respectively, while boric acid remains a guideline-recommended option for azole-resistant NAC infections. Multiple phase II–IV trials are actively evaluating probiotics, novel oral antifungals, and optimized delivery strategies, signaling a promising pipeline for future VVC management.

Ultimately, overcoming VVC requires a holistic, multidisciplinary strategy. The actionable implications of this review are threefold: (1) In diagnostics, we recommend implementing molecular methods (PCR, MALDI-TOF MS) and pH-adjusted antifungal susceptibility testing to guide species-specific, resistance-informed therapy. (2) In therapeutics, when azole resistance is confirmed or suspected (particularly for non-albicans *Candida*), clinicians should adopt non-azole alternatives—boric acid, nystatin, ibrexafungerp, or oteseconazole—rather than repeating empirical azole courses. (3) For future directions, research should prioritize biofilm-disrupting agents, probiotic microbiome modulation, and nanotechnology-based delivery systems as adjunctive strategies to reduce recurrence. These approaches, integrated with robust patient education and antifungal stewardship, can move the field from reactive treatment toward sustainable, precise management, thereby reducing the global burden of VVC.
